# ADVERSE MUSCLE COMPOSITION IS LINKED TO LOWER MITOCHONDRIAL RESPIRATION AND SLOWER WALKING SPEED

**DOI:** 10.1093/geroni/igae098.2010

**Published:** 2024-12-31

**Authors:** Sofhia Ramos, Katherine Glockner, Giovanna Distefano, Peggy Cawthon, Jennifer Linge, Olof Dahlqvist Leinhard, Bret Goodpaster, Paul Coen

**Affiliations:** AdventHealth, AdventHealth, Florida, United States; California Pacific Medical Center, San Francisco, California, United States; AdventHealth, Orlando, Florida, United States; California Pacific Medical Center, San Francisco, California, United States; AMRA Medical AB, Linköping, Ostergotlands Lan, Sweden; Linköping University, Linköping, Ostergotlands Lan, Sweden; Advent Health, Orlando, Florida, United States; AdventHealth, Orlando, Florida, United States

## Abstract

Low muscle mass and fat infiltration are core features of sarcopenic obesity. We examined skeletal muscle mitochondrial respiration and walking speed in older adults with normal muscle composition (NMC), only low muscle volume (LMV), only high fat infiltration (HFI) and the combination of LMV and HFI, (adverse muscle composition (AMC)). Muscle composition was determined by whole body MRI and high resolution respirometry was performed on muscle biopsies from N=828 participants (76.3 ± 5.0 years old, 59.4% female) in the Study of Muscle, Mobility and Aging (SOMMA). We found that mitochondrial respiration was significantly lower in individuals with LMV (-10%), HFI (-9%) and AMC (-8%), compared to those with NMC. In multivariate regression models, 4m and 400m walking speeds were significantly slower in LMV (400m: β=-0.10 4m: β=-0.07, both p< 0.05), HFI (400m: β=-0.14, 4m: β=-0.13, both p< 0.01) and AMC (400m: β=-0.19, 4m: β=-0.16, both, p< 0.01) groups, compared to NMC (400m: 1.11m/s, 4m:1.10m/s). Compared to NMC, mitochondrial respiration explained ~20% of the variance in 400m walking speed in LMV, ~64% in HFI, and ~47% in AMC, while all three groups remained significantly slower. Compared to those with NMC, mitochondrial respiration explained ~29%, and ~77% of the variance in 4m walking speed in LMV and HFI respectively, abolishing group differences in 4m walking speed, while mitochondrial respiration explained ~50% of the variance in ACM. In conclusion, low muscle volume, high fat infiltration and AMC phenotypes are linked to lower mitochondrial respiration and slower walking speed in older adults.

